# Authentic assessment meets sustainable development: Bringing meaning to undergraduate Physiology education

**DOI:** 10.1113/EP093236

**Published:** 2025-11-10

**Authors:** Mary McGahon, Sarah Geraghty, Clare Foy, Sean Roe

**Affiliations:** ^1^ Centre for Biomedical Sciences Education Queen's University Belfast Belfast Northern Ireland UK

**Keywords:** authentic assessment, contextual learning, meaningful education, Physiology education, sustainable development

## Abstract

Sustainable development is a growing global concern, but university students in scientific fields such as Physiology struggle to see its direct relevance to their studies. This research explores how an authentic assessment can integrate the United Nations (UN) sustainable development goals (SDGs) into Physiology education. Students were tasked to design and present a group poster on the connections between an SDG and Physiology. Through a mixed‐methods approach utilizing questionnaires, pre‐ and post‐assignment data were collected and the students’ perceptions of the links and learning opportunities explored. Students reported a shift in how they perceived the intersection between Physiology and societal issues, with many expressing a newfound passion for sustainability. Awareness and understanding of the SDGs increased significantly (19% and 36%, respectively). Students made connections between Physiology and goals such as No Poverty (SDG1), Quality Education (SDG4), Gender Equality (SDG5), and Climate Action (SDG13). Thirteen of the 17 SDGs were rated as significantly more relevant to Physiology after the assignment, among these, several less obviously related goals like Affordable and Clean Energy (SDG7), Sustainable Cities and Communities (SDG11), Responsible Consumption and Production (SDG12), and Partnership for the Goals (SDG17). The remaining four (SDGs 2, 3, 6 and 15) had high initial relevance ratings, which did not significantly change, serving as an internal control supporting the validity of the observed increases for other goals. Responses to open questions suggested that the students’ experience of the assessment was rich in context and meaning, making Physiology more than ‘just being a uni topic’.

## Abstract (highlights)

- **What is the central question of this study?**
  How can integration of UN sustainable development goals (SDGs) into Physiology education enhance students’ awareness and understanding of their relevance?
- **What is the main finding and its importance?**
  Students’ awareness and understanding of the SDGs increased significantly, with many making meaningful links and deeper connections between Physiology, societal issues and global challenges. This highlights the value of authentic assessment in fostering interdisciplinary thinking and sustainability engagement in science education.

## 1 INTRODUCTION

### 1.1 The United Nations sustainable development goals and higher education

The United Nations Sustainable Development Goals (SDGs) were introduced in 2015 to provide a comprehensive framework for fostering global economic prosperity, social equity and environmental sustainability. The 17 goals address issues such as poverty, hunger, justice, health, gender, peace, sustainability and climate – issues arguably central to continued human survival on the planet (United Nations, n.d.). Unfortunately, however, the SDG 2025 progress report indicates that only 18% of these targets are currently on track, calling for urgent action across diverse sectors, not least of which is education (United Nations, 2025).

This deficit is increasingly being recognised in higher education institutions, which are playing a fundamental role in driving sustainable development by integrating SDGs into their curricula. Education for Sustainable Development (ESD) underscores the interconnectedness of health, ecological stewardship and societal well‐being (UNESCO, n.d.). Many academic institutions have embedded ESD within their strategic frameworks, emphasising social responsibility and sustainable decision‐making among students. Our own institution places ESD front and centre in its ‘Strategy 2030’ (Queen's University Belfast, 2024), and Advance HE – the UK national body promoting the effectiveness, impact and quality of higher education teaching – has also positioned ESD as central to its mission (Advance HE, 2025).

### 1.2 The discipline of Physiology and the sustainable development goals

Recognising that ESD is central to survival and quality of life on the planet, Physiology learned societies have developed consensus statements on the UN SDGs (The Physiological Society, 2023a) and global heat resilience (The Physiological Society, 2024), while also formulating a society policy on sustainability (The Physiological Society, 2023b). It is evident that, as a discipline, Physiology recognises the gravity of these issues.

### 1.3 Physiology specific examples (applied SDGs)

With physiology directly concerning the function of the human body, SDG3 (Good Health and Well‐being) explicitly relates to the subject. In addition, many of the other SDGs significantly influence normal physiology. Understanding the physiological mechanisms connecting nutrition with neural development highlights the urgent need for equitable access to adequate nutrition to support cognitive development and learning success, as emphasised in SDG2 (Zero Hunger) and SDG4 (Quality Education) (Andersson et al., 2012; Bernal, 2007; Pedraza et al., 2006). Similarly, SDG5 (Gender Equality) highlights disparities in healthcare and research faced by women and gender‐diverse individuals through systemic health inequalities. These disparities manifest physiologically in, for example, lower levels of troponin I in females experiencing myocardial infarction, leading to under‐diagnosis (Shah et al., 2015), or non‐representative reference ranges for transgender individuals on gender‐affirming therapy (Greene et al., 2021). Economic stability, addressed by SDG8 (Decent Work and Economic Growth), also has profound physiological implications. Chronic occupational stress and job insecurity contribute to dysregulated hypothalamic–pituitary–adrenal (HPA) axis activity and can lead to sustained elevations in cortisol and an increased risk of cardiovascular diseases (Kim et al., 2018; Lawes et al., 2022). Conflict and displacement, linked to SDG16 (Peace, Justice and Strong Institutions), also pose significant physiological challenges exacerbated by climate change (as actioned in SDG13). Environmental degradation and resource scarcity linked to climate change drive mass migrations, leading to overcrowded and unsanitary living conditions. These give rise to the spread of infectious diseases (Bharadwaj & Huq, 2022) and chronic stress, which dysregulates the HPA axis, elevating the risk of post‐traumatic stress disorder (PTSD), depression and metabolic disorders (Lawrence & Scofield, 2024). Strengthening institutional responses to climate‐induced displacement is crucial in reducing the physiological impact of climate‐related conflict. The Physiological Society of Great Britain and Ireland has recognised this with a 2025 conference on the physiology of heat stress and a 2024 consensus statement on global heat resilience (The Physiological Society, 2024).

Notwithstanding that almost every one of the 17 UN SDGs has a physiological correlate, these are often not immediately apparent, particularly to the early undergraduate student of Physiology or Life Sciences.

### 1.4 Strategies for effective learning and student engagement (context rich education and authentic assessment)

While it is apparent that the discipline of Physiology is now playing an increasing role in delineating and evidencing health issues surrounding the SDGs, there are myriad potential benefits to Physiology education flowing from this association. It has long been established that in Physiology, context‐rich ‘situational’ classes in which the student is invested result in deeper learning and more profound retention (Goodman et al., 2018; Richardson, 2008; Roe et al., 2024). The concept of context in life sciences education is expanded by Koens and colleagues, who stress a motivational component in any learning situation, which they term the ‘commitment dimension’ of educational context (Koens et al., 2005). Further, cognitive load theory posits that understanding and deep learning are predicated on incorporating learning schema using short‐term working memory into the less limited, more permanent stores of long‐term memory (Paas et al., 2003, 2010). In cognitive load theory, ‘extraneous’ cognitive load can limit learning by burdening the learner excessively in the acquisition phase. It is incumbent on educators to ensure that the ‘extraneous’ cognitive load incorporates what they term a ‘germane’ element that resources learners to deal with the incoming information. That germane element may be provided in well‐designed classes by encouraging creativity and imagination, simultaneously enhancing the motivation to learn and reducing the emotional burden of the learning task by making it meaningful. Since the actioning of SDGs particularly resonates with the younger generation that populates most Physiology or Life Science classes (Thomaes et al., 2023), application of the SDGs to Physiology provides a unique opportunity to contextualise learning and provide a cognitive load in Physiology classes and assessments which is germane rather than extraneous.

By linking global sustainability to Physiology and by including the elements of self‐directed, autonomous learning, students have the potential to engage in assessments which authentically support the development of real‐world competencies, ethical awareness, and interdisciplinary thinking. Furthermore, incorporating SDGs into Physiology education has the potential to promote critical thinking, evidence‐based problem‐solving and prepare students to tackle real‐world global issues. Understanding the physiological implications of climate change, pollution and urbanisation may enable students to devise informed strategies to mitigate associated health risks. Aligning Physiology education with SDGs also has the potential to enhance interdisciplinary collaboration, bridging connections between public health, environmental sciences and policymaking (Kioupi & Voulvoulis, 2019).

To harness the connection between Physiology and SDGs, thus engaging students in meaningful, authentic assessment, we devised an innovative poster assignment. This challenged students to draw links between Physiology and one of the 17 SDGs (or one of the nine protected characteristics as defined by the UK Equality Act 2010) (UK Government, 2010). Before and after the assignment, we gathered student feedback through questionnaires that included both numerical evaluations (self‐reported Likert scores) and open‐ended responses about their experience of the process.

## 2 METHODS

### 2.1 Ethical approval

This study conformed to the standards set by the *Declaration of Helsinki*, except for registration in a database. Ethical approval for the study design, assessment and distribution of questionnaires was granted by the Queen's University Belfast School of Medicine, Dentistry and Biomedical Science Research Ethics Committee (MHLS 23_144). Questionnaires were completely anonymous and, as stated on the questionnaire information sheet (informing the participant of their rights and purpose of the study), submission of a completed questionnaire by an individual was voluntary and would be accepted as implied consent. Students were informed that any results would be used for educational research only and were not a means of student assessment. It was also made clear that students were under no obligation to complete questionnaires, giving them the automatic right to withdraw at any point during the process.

### 2.2 Design of the assignment, participants and data collection

After answering a pre‐assignment questionnaire, second‐year Queen's University Belfast (QUB) Human Biology students completed a curriculum‐embedded group assignment, creating posters on self‐selected physiological topics which they needed to link to relevant SDGs. Students could alternatively choose to complete their assignment by relating Physiology to an aspect of Equality, Diversity or Inclusion (EDI) as defined by the protected characteristics of the UK Equality Act 2010 (UK Government, 2010) – a parallel aspect of the assignment but one which is not reported upon further in the present paper. Students were given a basic introduction to SDGs and protected characteristics (i.e. a list of the 17 SDGs and nine protected characteristics, as well as two examples of how SDGs or EDI could influence Physiology, such as global warming and thermoregulation for SDG13, and fasting during Ramadan for Religion or Belief). Students worked in small groups (ideally three members, with a maximum of five) to conduct both individual and collaborative research, develop a poster and deliver a live presentation to their peers approximately 5 weeks after the initial assignment introductory session. Each presentation lasted 10 min and was followed by a 5‐min student‐led question‐and‐answer session. Free periods were timetabled for creation of the poster closer to the submission deadline, and one additional 1‐h session was held to support the development of communication (written/graphic/oral) skills in the intervening period. Each group peer‐assessed and provided feedback for one other group, and the presentation was co‐marked by two to three academics with marks weighted 20%:80%, respectively. The assignment contributed 20% to the overall module mark. Marks from peers were in good accordance with those from academics; however, where peer marks did not equate to standards set by the QUB undergraduate conceptual marking scale for the level of learning (Queen's University Belfast, 2022), the option to moderate was available to the module convenor, who therefore had the final say in the outcome mark of the assessment.

### 2.3 Questionnaires

To assess impact, students completed pre‐ and post‐assignment questionnaires (33 questions pre‐assessment and 34 post‐assessment) on SDG awareness (‘On a scale of 1–10 [1 not at all aware, 10 highly aware] rate your awareness of Sustainable Development Goals [SDGs]’), understanding (‘On a scale of 1–10 [1 no understanding, 10 full understanding] rate your understanding of Sustainability Development Goals [SDGs]’, ‘Comment on your awareness and understanding of Sustainability Development Goals [SDGs]:’), and relevance to Physiology (‘What physiological effects relating to SDGS are you aware of?’ and ‘Rate your level of agreement [Strongly disagree, Disagree, Neither agree nor disagree, Agree, or Strongly agree] that Physiology is relevant to SDG X’ (each SDG is listed by name and number). One additional question post‐assignment (‘Comment on how or if this assignment is likely to impact your future behaviours regarding SDGs’), thus providing reflections on their learning. Because they included semi quantitative Likert score questions and invitations to write open commentary on the classes, this mixed methods approach allowed both quantitative and qualitative analysis of the classes.

### 2.4 Quantitative analysis of the data

Quantitative analysis of the classes was evaluated by questions in which the Likert scale was employed. Self‐reported awareness and understanding of SDGs was rated from 1 (no awareness/understanding) to 10 (full awareness/understanding). In the case of how relevant students considered each SDG to physiology, scores were rated from 1 (not relevant/strongly disagree) to 5 (highly relevant/strongly agree). Differences in Likert scores between questionnaires completed before and after the assignment could then be tested for statistical significance on GraphPad Prism (GraphPad Software, Boston, MA, USA) using one‐way analysis of variance (ANOVA; Kruskal–Wallis) followed by Dunn's multiple comparisons *post hoc* test between appropriately selected groups. In each case *P *< 0.05 was considered statistically significant. Results are reported on graphs as means ± standard deviation (SD) with further information (SEMs, CIs and exact *P*‐values) provided in Table 2 and within the Appendix (Tables A1 and A2). An overall response rate of approx. 84% (from 61 student polled) was achieved in this study.

### 2.5 Qualitative analysis of the data

Because the questionnaires included invitations to openly comment upon the lived experience of engaging in the assignment, thematic analysis could be used to discover, interpret and report meaningful patterns within the data (Pope et al., 2000). This involved systematically working through the statements from the open commentary using NVivo software to identify key themes that informed the discussion.

## 3 RESULTS

### 3.1 Awareness and understanding

Forty‐eight of 61 students polled responded within free text commentary regarding previous exposure to SDGs. Four students reported previous insight through secondary school level Geography classes and 21 specifically mentioning exposure gained during previous university related activities or assignments (particularly Carbon literacy training or first year assessments). Thirty students reported ‘some’ prior knowledge: ‘*Heard of them from university but don't have an in depth understanding of them*.’ And ‘*I am aware of them as I was taught them in first year; however, I have forgotten them. Understanding is limited*.’ Thirteen students expressed a more ‘solid’ understanding: ‘*They try to make their world a better place, goals for countries, universities, companies and schools to work towards*.’ And: ‘*It allows the world to become a better place. Allows the countries to set goals on how to make their country better for the population*.’ Five reported little or no previous awareness/understanding of SDGs prior to the introduction of this assignment with comments such as: ‘*I don't know what SDGs are*.’

**FIGURE 1:**
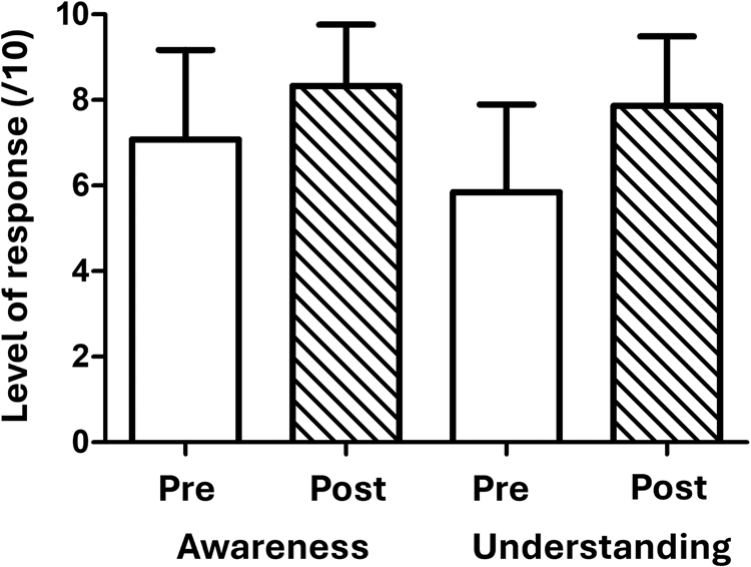
Making and presenting posters in the context of physiology increases awareness and understanding of SDGs. Student reported Likert scored responses out of 10 for awareness and understanding of SDGs pre‐ and post‐assignment. *n* = 51 pre‐ and 52 post‐assignment (mean ± SD). One‐way ANOVA (Kruskal–Wallis test; *P *< 0.0001) with Dunn's multiple comparisons *post hoc* test (*P* = 0.0007 and *P *< 0.0001 for awareness and understanding tested pre‐ versus post‐assignment, respectively; *P* = 0.0116 and *P* = 0.74 for awareness versus understanding pre‐ and post‐assignment, respectively. Further breakdown of data provided in Appendix Tables A1 and A2).

To explore this quantitatively we asked students to rate their awareness and understanding using a 10‐point Likert scale with 1 being no awareness or understanding and 10 being highly aware/having full understanding. Pre‐assignment, students reported higher scores for awareness over understanding with a substantial rise in understanding identified post‐assignment. On average, scores for student awareness of the SDGs increased by 19% from 7.02 ± 2.07/10 (mean ± SD) pre‐assignment (prior to the introduction to the assignment) to 8.33 ± 1.44/10 post‐assignment (after the entire class had presented their posters; *n* = 51 pre‐assignments, *n* = 52 post‐assignments; *P* = 0.0013; Figure 1, Appendix Tables A1 and A2). Scores for understanding of SDGs showed a 36% increase, from 5.78 ± 2.02 to 7.87 ± 1.62/10 (*P *< 0.0001). While a significant difference in awareness vs. understanding was detected pre‐assignment this difference was not present post‐assignment (*P* = 0.0116 and 0.74, respectively; Appendix Table A2).

### 3.2 Topics related to SDGs presented by students

**FIGURE 2:**
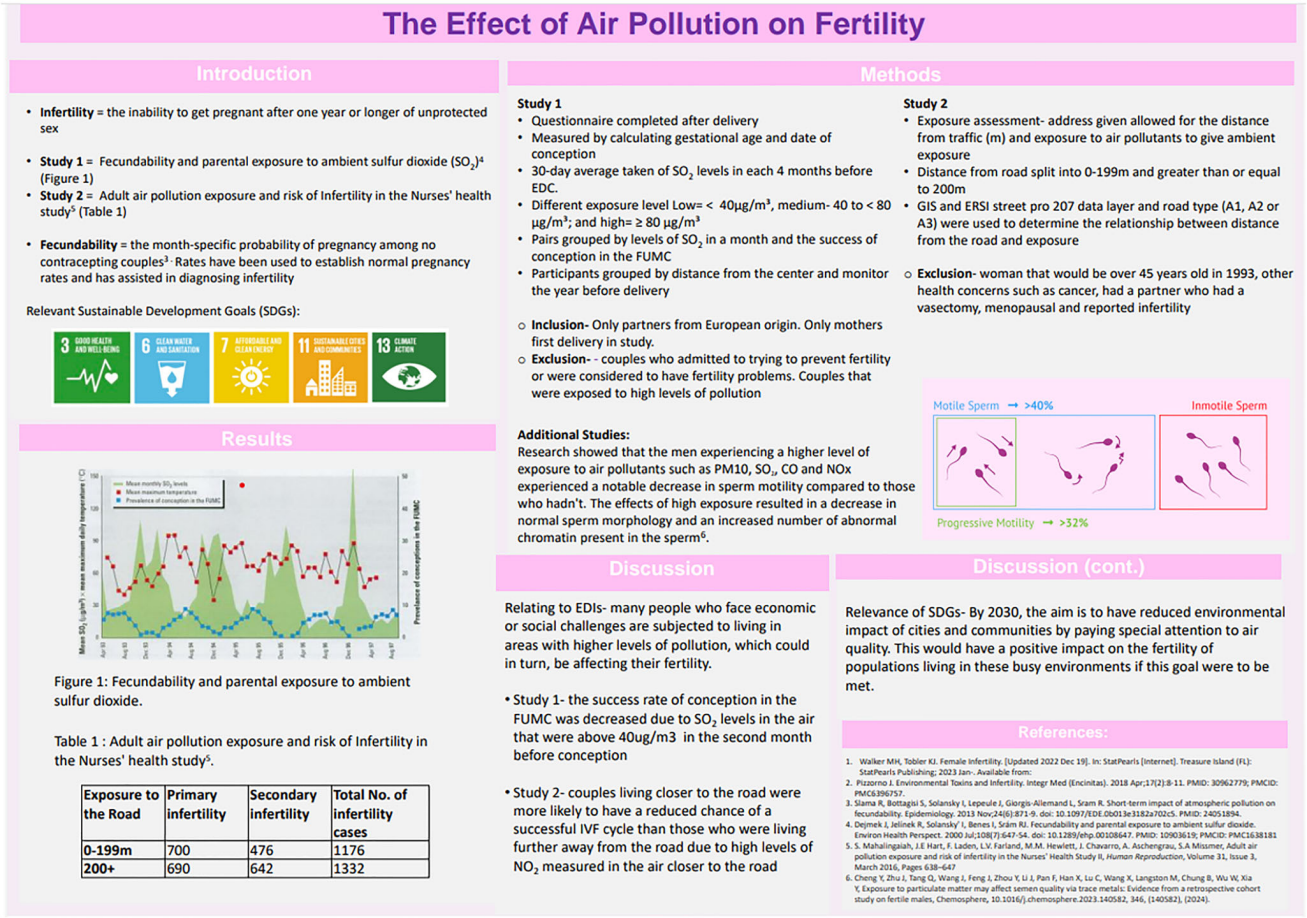
Example of student designed poster linking SDGs 3, 6, 7, 11 and 13 to reproductive physiology.

**FIGURE 3:**
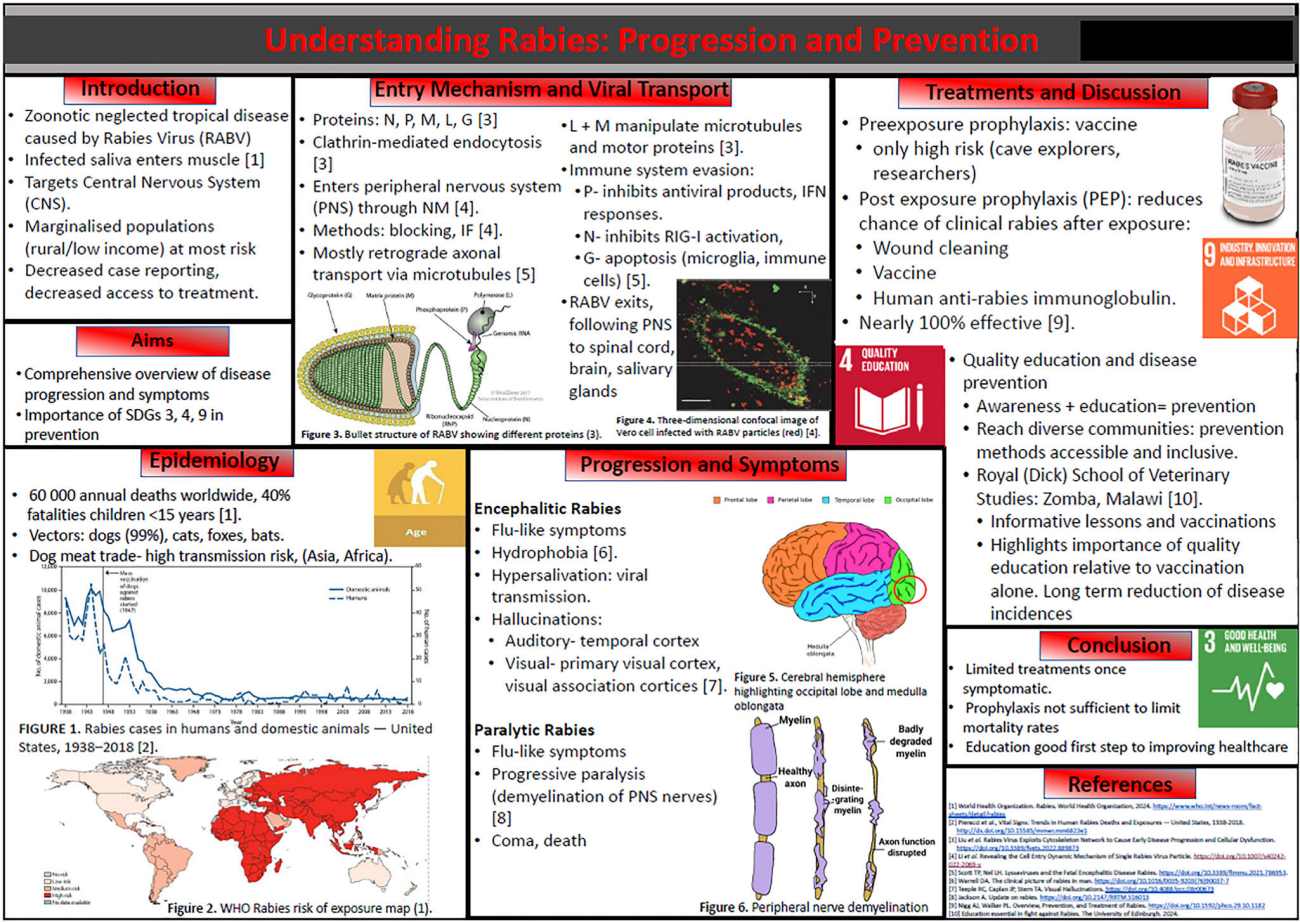
Example of student designed poster linking SDGs 3, 4 and 9 to immune physiology.

**FIGURE 4:**
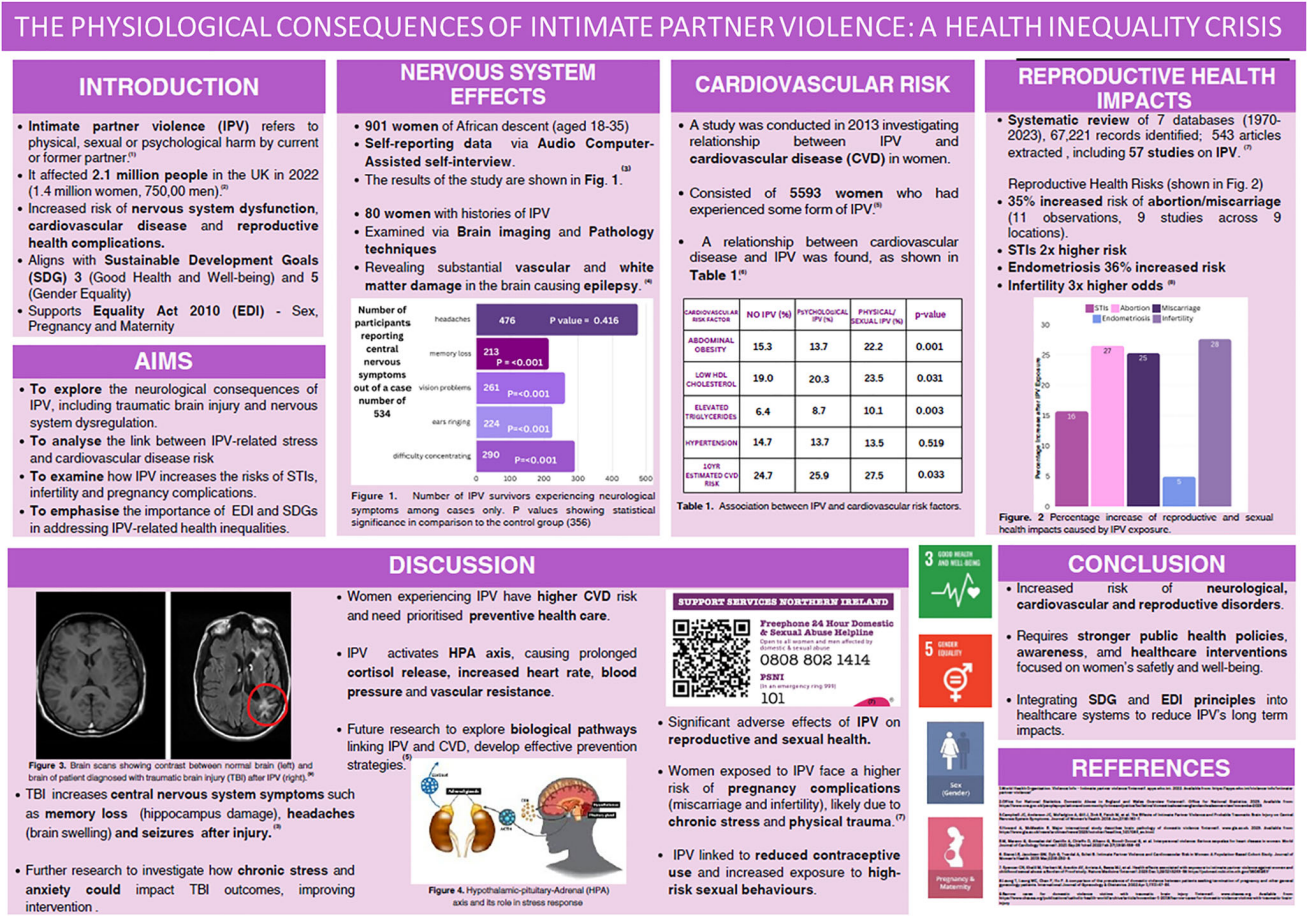
Example of student designed poster linking SDGs 3 and 5 to brain, cardiovascular and reproductive physiology.

**FIGURE 5:**
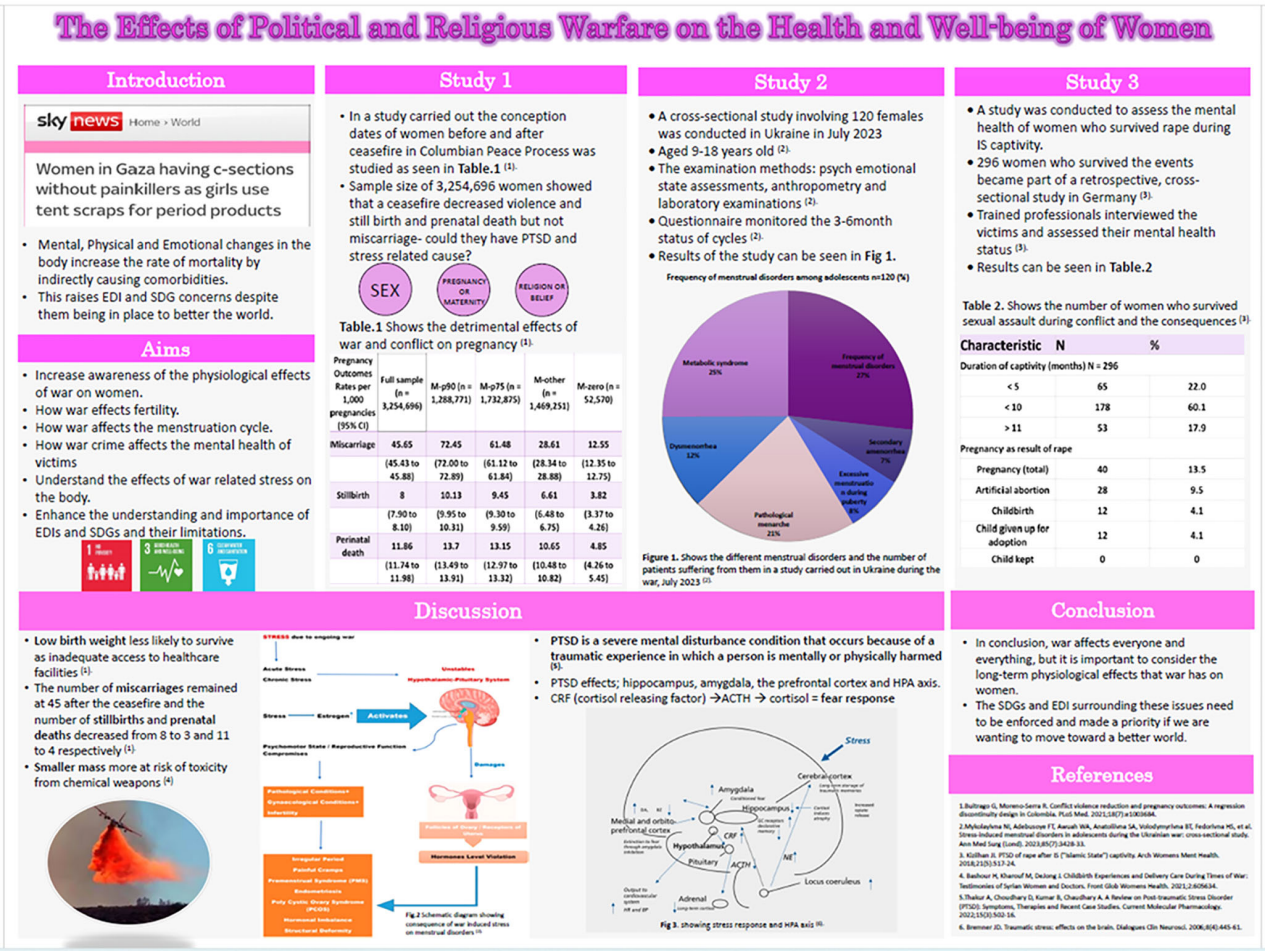
Example of student designed poster linking SDGs 1, 3 and 6 to brain and developmental physiology.

Over the course of two academic years, 18 posters were presented which collectively students related to 13/17 SDGs (Table 1). The topics presented generally fell into one or more of six categories: (1) the effects of pollution, (2) gender disparities in healthcare, (3) disability, violence and trauma, (4) gut health, (5) marine health, and (6) communicable diseases, which were related to respiratory, cardiovascular, muscle, gastrointestinal, neural, immune, reproductive and developmental physiology. Examples of the posters are reproduced (anonymised and with student permission) in Figures 2, 3, 4, 5. SDG3 (Good Health and Well Being), SDG5 (Gender Equality), SDG10 (Reduced Inequalities) and SDG13 (Climate Action) were the most frequent SDGs identified by students as directly relating to their chosen aspect of Physiology. Students failed to directly link SDG8 (Decent Work and Economic Growth), SDG12 (Responsible Consumption and Production), SDG15 (Life on Land) and SDG17 (Partnership for the Goals) to their poster topics. During post‐presentation questions and discussions, academics were able to identify and explore with students the potential link to all of the remaining SDGs on the basis of their presentations.

**TABLE 1:** Posters presented by two cohorts of human biology students from Queen's University Belfast and their relationship to the UN Sustainable Development Goals.

Poster title	Cohort (year group)	Related SDGs identified by students	Additional SDGs identified by academics
The physiological impact of air pollution on respiratory health	2024/25	3, 13	7, 11, 12
Oxygen inequality: How pollution and climate change affect global respiratory health.	2024/25	3, 11, 13	7, 12
The effect of air pollution on fertility	2023/24	3, 6, 7, 11, 13	12, 17
Hormonal fluctuation differences in men and women: impact on work productivity	2023/24	5	8, 10
Are women equally represented in clinical trials?	2023/24	3, 5, 10	
The effects of political and religious warfare on the health and well‐being of women	2023/24	1, 3, 6	16
The disparities in women's healthcare research: addressing the issue	2024/25	5	2, 3
ADHD: Presentation differences in males and females	2023/24	^*^	5
How music can positively impact reading development dyslexia	2023/24	4, 10	1
Sound solutions: music therapy's role in advancing mental health	2024/25	3, 10	17
The physiological impact of trauma	2023/24	2	1, 16
The physiological consequences of intimate partner violence: a health inequality crisis	2024/25	3, 5	16
Are you born violent? Comparing genetic markers and environmental factors in determining violence and aggression	2024/25	16	5
The microbiome and autism spectrum condition: exploring the gut–brain axis	2024/25	3, 10	17
Food for thought: The ultra‐processed food pandemic	2023/24	1, 3	2
Feminization of male fish	2023/24	6, 14	5
The unique splenic evolution of the Bajau people	2023/24	13, 14	6
Understanding rabies: Progression and prevention	2024/25	3, 4, 9	15

*Note*: ^*^Authors of poster entitled ‘ADHD: Presentation differences in males and females’ solely identified links to EDI protected characteristics; thus, they still met the criteria for assignment, which allowed for alignment with either an SDG or EDI related topic.

### 3.3 Student perception of SDG relevance to Physiology

**FIGURE 6:**
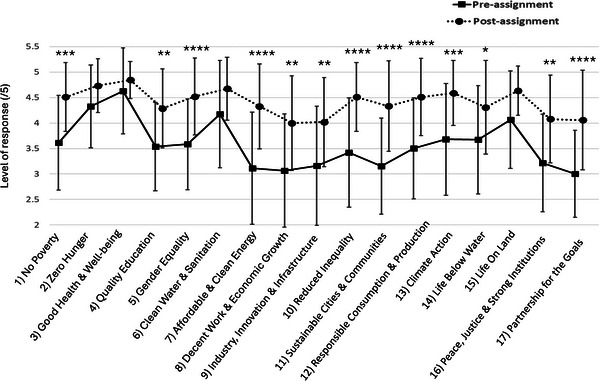
Making and presenting posters relating SDGs to Physiology increased students’ perception of the relevance of specific SDGs to Physiology. Student reported Likert scored responses rated 1–5 for perceived links of individual SDGs to Physiology pre‐ and post‐assignment (mean ± SD). *n* = 45–48 pre‐assignment and 51–52 post‐assignment. One‐way ANOVA (Kruskal–Wallis; *P *< 0.0001) with Dunn's multiple comparisons *post hoc* tests (indicated on graph as *P < 0.05, **P < 0.01, ***P < 0.001, ****P < 0.0001; further breakdown of data and exact *P*‐values provided in Table 2).

A statistically significant increase in perceived relevance of SDGs to Physiology was also observed for 13/17 SDGs. On average, students' ratings increased from 3.58 ± 0.98/5 pre‐assignment to 4.41 ± 0.74/5 post‐assignment. Significant differences (Figure 6, Table 2; *P *< 0.05) were observed in the perceived relevance of 13 of the SDGs to Physiology, including SDG1 (No Poverty), SDG4 (Quality Education), SDG5 (Gender Equality), SDG7 (Affordable and Clean Energy), SDG8 (Decent Work and Economic Growth), SDG9 (Industry, Innovation and Infrastructure), SDG10 (Reduced Inequalities), SDG11 (Sustainable Cities and Communities), SDG12 (Responsible Consumption and Production), SDG13 (Climate Action), SDG14 (Life Below Water), SDG16 (Peace, Justice and Strong Institutions) and SDG17 (Partnership for the Goals). Only four of the SDGs did not show a significantly higher student reported relevance to Physiology pre‐ versus post‐assignment (SDG2 – Zero Hunger, SDG3 – Good Health and Well‐being, SDG6 – Clear Water and Sanitation and SDG15 – Life on Land; Table 2; *P *> 0.05). It is noteworthy that all four of these SDGs already scored high on the Likert scale for student perceived relations to Physiology prior to the introduction of the assignment (mean score >4/5) thus students were already aware of the link between these SDGs and Physiology and as such undertaking of the assignment would be expected to have a lessor or no impact in these areas specifically. This lack of significance could be regarded as an internal control, suggesting that the positive findings regarding other SDGs represent true recognition of the connections between Physiology and sustainable development and therefore novel learning.

**TABLE 2:** Summary data and statistical analysis of student perceived relevance of physiology to SDGs pre‐ and post‐assignment.

	Pre‐assignment				Confidence intervals (95%)		Post assignment				Confidence intervals (95%)		Dunn's multiple comparisons test
SDG no. and name	Mean /5	SD	SEM	N	Lower	Upper	Mean /5	SD	SEM	N	Lower	Upper	Adjusted *P‐*value
(1) No poverty	3.609	0.930	0.137	46	3.33	3.88	4.510	0.674	0.094	51	3.48	6.93	0.0004
(2) Zero hunger	4.319	0.810	0.118	47	4.08	4.56	4.731	0.528	0.073	52	3.7	7.13	0.9612
(3) Good health and well being	4.625	0.841	0.121	48	4.38	4.87	4.846	0.364	0.051	52	3.81	7.23	>0.9999
(4) Quality education	3.533	0.869	0.129	45	3.27	3.79	4.288	0.776	0.108	52	3.27	6.74	0.0061
(5) Gender equality	3.578	0.892	0.133	45	3.31	3.85	4.519	0.754	0.105	52	3.49	6.95	<0.0001
(6) Clean water and sanitation	4.170	1.049	0.153	47	3.86	4.48	4.673	0.617	0.086	52	3.64	7.08	0.6342
(7) Affordable and clean energy	3.109	1.100	0.162	46	2.78	3.44	4.327	0.834	0.116	52	3.31	6.78	<0.0001
(8) Decent work and economic growth	3.064	1.111	0.162	47	2.74	3.39	4.000	0.929	0.129	52	0.538	10.1	0.0027
(9) Industry, innovation and infrastructure	3.156	1.167	0.174	45	2.8	3.51	4.019	0.874	0.121	52	2.78	15.3	0.0052
(10) Reduced inequality	3.417	1.069	0.154	48	3.11	3.73	4.510	0.674	0.094	51	−0.054	6.5	<0.0001
(11) Sustainable cities and communities	3.152	0.942	0.139	46	2.87	3.43	4.333	0.887	0.124	51	3.77	9.31	<0.0001
(12) Responsible consumption and production	3.500	0.989	0.143	48	3.21	3.79	4.510	0.758	0.106	51	4.01	9.69	<0.0001
(13) Climate action	3.674	1.097	0.162	46	3.35	4.00	4.588	0.638	0.089	51	3.59	6.91	0.0008
(14) Life below water	3.667	1.059	0.153	48	3.36	3.97	4.308	0.919	0.127	52	3.29	6.76	0.0460
(15) Life on land	4.063	0.954	0.138	48	3.79	4.34	4.635	0.486	0.067	52	3.61	7.04	0.1960
(16) Peace, justice and strong institutions	3.213	0.954	0.139	47	2.93	3.49	4.077	0.860	0.119	52	3.06	6.55	0.0026
(17) Partnership for the goals	3.000	0.853	0.127	45	2.74	3.26	4.058	0.978	0.136	52	3.04	6.54	<0.0001

### 3.4 Qualitative analysis of student responses

Thematic analysis identified four themes within student responses:

1. Increase in awareness and understanding. Twenty‐four students across both year groups reported that their awareness and understanding of the SDGs improved following the assignment. Many were previously unaware of SDGs or had only limited knowledge from prior school experience (e.g., A‐level Geography). One student reflected, ‘*I wasn't well‐educated on the SDGs before, but I am more aware now*,’ while another noted, ‘*I learnt about the SDGs through GCSE and A Level Geography, so I have a good understanding*.’ The breadth of information covered was alluded to by several respondents who said they gained information on ‘*sex, pregnancy, infertility, miscarriage. How access to clean water + sanitation can affect the physiology of women + men – life expectancy. How different religions practices can affect physiology*’ and ‘*So many* [topics] – *autism and gut/brain axis, respiration in global warming, CVD affects [sic] from abuse*’. Students described becoming more aware of the relevance of SDGs to real‐life issues, particularly in relation to physiology, health inequalities and environmental impacts: ‘*This assignment has greatly helped my understanding of the SDGs and how much they apply to real‐life situations and science*.’ Several recognised that more SDGs apply to physiology than previously realised: ‘*I've seen from this assignment how a lot more SDGs are physiology‐related than I originally thought*.’
2. Future behavioural intentions. Eighteen students also reported a greater/new sense of advocacy/responsibility, while nine expressed the desire to educate others or explore SDG‐related topics further: ‘*I would also encourage females to take part in future clinical trials*.’ Others committed to behavioural changes: ‘*Recycle more – try to prevent too much of my own carbon footprint*,’ and ‘*I will be more aware of the health implications of ultra‐processed foods*.’ Students also linked physiology to global inequalities: ‘*I have more of an understanding of the impact of pollution on other people's culture and way of life, like the Bajans*.’ Students described thinking differently about how physiological knowledge intersects with societal issues. For instance: ‘*I have gained more understanding of how physiological changes are affected by inequality and world issues*.’ Others stated that they were now ‘*more likely to educate others on these issues*.’
3. Meaning in the academic, professional and personal domains. Fourteen students reported changes in how they regarded the importance of SDGs to their future education, research and careers: ‘*By being provided with information on SDGs and EDI … I wish to use the information to drive future investigation*.’ Others reflected: ‘*Definitely have a better understanding now and feel able to include them in future assignments and actions*’ and ‘*it made me much more aware of the widespread and personal implications of crisis in the world*’. Other students contended that coverage of the SDGs should be seen ‘*not just as a box to tick off, but as a framework to translate lecture material into real‐world applications*’ and that ‘*physiology was not just about homeostatic mechanisms, regulation and biological systems but also about resilience, inequality, and human wellbeing in a global context*.’ These quotes evidence an increasing appreciation among students of the relevance of the subject to both academic pursuits and personal actions, pertaining ultimately to global issues. Additionally, the opportunity to discuss topics that were important in a safe space among peers allowed students to explore emotionally and politically charged aspects of life without judgement. With the focus being on physiological effects rather than moral judgments or ideological positions, students felt able to open up. This is also reflected in some of the contentious subjects that students chose to relate to the SDGs, examples being posters on ‘The physiological consequences of intimate partner violence’, and ‘The effects of political and religious warfare on the health and well‐being of women’ (Figures 5 and 6). The conclusion sections of many of posters read effectively as a call to activism. From the poster on Political and religious warfare: ‘*In conclusion … it is important to consider the long term physiological effects that war has on women. The SDGs and EDI surrounding these issues need to be enforced and made a priority if we are wanting to move toward a better world*.’ From the poster on intimate partner violence (IPV): ‘*IPV causes increased risk of neurological, cardiovascular and reproductive disorders. It requires stronger public health policies, awareness and healthcare interventions focused on women's safety and well‐being. Integrating SDG and EDI principles into healthcare systems …* [is required] … *to reduce IPV's long term impacts*’.
  The profound power of the assessment to prompt attitudinal and culture change is also evidenced in commentary written by students after the assignment. Reflecting after the assignment one participant commented that ‘*opening up in a safe space hasn't just improved the work – it has expanded my sense of what can be*’, and from another ‘*by working together, each contributing something distinct and allowing ourselves to be vulnerable we were able to gain confidence and produce a higher standard of work … I learned that my initial insecurities were much less important than my willingness to engage*.’
  The evidence suggests that our assessment strategy created an environment conducive to professional development, critical thinking and deeper understanding, enabling students to reflect on complex global issues through a scientific lens. Student testimony also suggests that they derived great personal meaning from the experience of creating, presenting and defending the posters. ‘*It really has opened my eyes to how physiology can relate so much to the wider world instead of just being a uni topic*.’
4. Barriers to integration of SDGs into the curriculum. Three students expressed reservations finding SDG content burdensome or detached from their core studies: ‘*Unless I have to do a similar project, it won't affect it*,’ and ‘*SDG information is seen as a burden (compulsory Carbon Literacy training) but it's good to be aware of them to better the planet*.’ Nevertheless, students generally appreciated the real‐world connections made through SDGs and were more receptive when SDGs were tied directly to physiology: ‘*Definitely kept in mind for future science and physiology*.’

## 4 DISCUSSION

### 4.1 Salient results

The results suggest that this assignment significantly improved students’ awareness of the SDGs in general and how they relate to physiology in particular. It is also evident from testimony that students have been imbued with a sense of purpose and meaning after the project, and that the experience of interacting with each other in the creation and presentation of the posters was deeply meaningful. Many left the class with a sense of mission and purpose. While some of these outcomes were what we had hoped to observe, students achieved far beyond the initial expectations of the investigators. In these emergent phenomena lie much of the value of the study.

### 4.2 Authentic and powerful physiology assessment

Authentic assessments such as those outlined here move beyond the realm of rote learning to encourage problem‐solving and the application of knowledge with meaning, reflection and collaboration. Integrating SDGs into the Physiology curriculum has the potential to allow students to make connections to human health and physiological processes through deeper (self‐directed) learning, critical thinking and enhanced student engagement (Koh, 2017). By designing assessments which allow students to make their own decisions about what they want to study, we are empowering them to determine their own pathway through the literature (akin to intrinsic learning), replicating what they would experience in postgraduate studies or a research position, thus preparing them for their next steps in scientific practice (Tang et al., 2024). By providing meaningful context, cognitive engagement with physiological concepts is thus increased and greater student buy‐in achieved (Höffken & Lazendic‐Galloway, 2024). Because students saw themselves in the assignment, they engaged far more with it than with any other within the module – or indeed within the year across the Human Biology programme, as indicated in end‐of‐module student experience surveys.

Another aspect of the work is the myriad rewards of engaging in peer‐to‐peer assessment, feedback and learning. Students engaging in peer assessment have been shown to have increased meta‐cognitive skills, reflexivity and emotional intelligence (Hay & Mathers, 2012). There is deep value to be gained from critiquing others’ work and then applying that critical lens back onto oneself. Building on this autonomy, a natural progression of this work would be to include an aspect of reflection. This internal feedback loop would not only enhance academic outcomes but also foster emotional intelligence and deeper personal investment in learning (Nicol, 2020).

### 4.3 SDGs and enhanced relevance in physiology education: physiology as activism?

One of the most striking observations from this study is how students made significant connections between SDGs and Physiology. When given the space to explore and direct their own learning, the links made and the deep dives they pursued proved profound, nuanced and well evidenced. The subject matter freely chosen by the students also reflects their deep concerns and enables them to interrogate, dissect and scientifically audit these concerns. Student expressions of future intention and behaviour change detailed in the results section can be regarded as evidence of student engagement and a deeper appreciation for the interdisciplinary nature of physiology. Consequently, this assessment has the potential to enhance the relevance of their education as demonstrated by the ‘call to activism’ in many of the posters’ conclusion sections. Given the potential to increase reflective practice and advocacy within students through this work, the benefits for student growth would easily offset the resistance to curricular change we describe below. Authentic classes and assessments like these have the potential to create passionate physiology activists, appropriate for this time of global climate change and political instability.

### 4.4 Emergent phenomenon: physiology as therapy?

A surprising and welcome theme apparent from the student commentary was that the experience of meeting, discussing, researching and presenting the posters was profoundly meaningful to the students. Students participated in animated discussions and brainstormed ideas readily. They collaborated not only on which topic to cover but also in researching and presenting it in the form of a compelling and cohesive argument, backed up with scientific literature and visually arresting posters (Figures 2, 3, 4, 5). As previously mentioned, many students chose topics which were contentious in nature and emotionally taxing to explore (e.g. the physiological consequences of intimate partner violence; the effects of political and religious warfare on the health and well‐being of women). Indeed, the students who presented on Intimate Partner Violence suggested that the module convenors provide links to support services for colleagues affected by these issues – suggestions immediately acted upon by the authors. Ultimately, students became ‘active agents’, taking ownership and developing passion regarding the intersectionality of sustainability and physiology, a task which is not easily achieved in conventional assessment modalities (Zimmerman, 2002).

This counteracts the generally held belief that STEM programmes are emotionally sterile (Seymour & Hunter, 2019). By allowing students to explore how sustainability impacts their body functions, we are providing them with the opportunity to discover where they personally fit into the curriculum, giving them a greater sense of belonging and scientific identity. By embracing the affective domain and harnessing the feelings that arise during this assignment, we have the potential to evoke an emotional response such that they are doing more than merely learning the literature. They are also becoming invested in understanding their body, the world in which they inhabit, and how they interact (Tea & Ovid, 2024).

The implications of this emotional engagement, however, run far deeper than changing perceptions of STEM subjects. A growing mental health crisis among undergraduate students has been acknowledged since at least 2018 (Pereira et al., 2019) and has been profoundly exacerbated by the COVID pandemic (Lewis & Stiebahl, 2025). Groups led by Bruce Hood at the University of Bristol and Laurie Santos at Yale (Hobbs et al., 2022, 2024) have proposed psychoeducational ‘well‐being’ courses as a means of addressing this crisis. These courses focus on enhancing both the hedonic (attainment of pleasure, happiness and life satisfaction) and eudaimonic (experience of meaning, growth, purpose and authenticity) aspects of well‐being. The ‘happiness hacks’ suggested in these courses reflect the principles of positive psychology described by Martin Seligman (2018). He posited that the foundation of well‐being could be summarised in the acronym PERMA (Positive Emotion, Engagement, Relationships, Meaning, Accomplishment). All of these investigators stress the importance of meaning and community, feeling part of something bigger than oneself. Hood (2024) encapsulates this nicely in contrasting ‘allocentricity’ as opposed to ‘egocentricity’ as the path to greater well‐being. The concept of meaning is further expanded in the work of Emily Esfahani Smith, who characterises four ‘pillars’ of meaning – belonging, purpose, storytelling, transcendence (Smith, 2017).

Many of these elements of well‐being and meaning are embedded within the assignment, and there is ample evidence for this in the student testimony detailed in the Results section, some of which details profound shifts in attitudes, cultural‐ and self‐awareness. Community, relationships, belonging and allocentricity all flow from the groupwork nature of the project and peer marking. The challenging subject matter with global implications provides the meaning and purpose. Transcendence, storytelling and accomplishment were evident in the poster presentation session, which was conducted in an upbeat, supportive atmosphere, resulting in some visually arresting posters that evidently involved both creativity and hard evidence. While not formally surveyed, the authors also experienced this as meaningful and joyful – one of the more fulfilling assessments undertaken during the academic year, and definitely conducive to good academic mental health; not to be undervalued.

### 4.5 Challenges: The need for personal, local and global culture change

While the present work suggests there is growing student support for integrating the SDGs into Physiology education, significant opposition remains both within academic circles and in the wider world. Globally, the integration of the SDGs into higher education is on the rise, particularly in disciplines like engineering, business, and social sciences (PRIME UN Initiative, 2018). However, the focus varies depending on the income level of the country. High‐income countries tend to focus on the theoretical aspects of the SDGs, while low‐ and middle‐income countries emphasise practical, real‐world applications. For these countries, the integration of SDGs into education can be seen as a secondary priority, as they may face more immediate challenges such as meeting basic educational standards and addressing fundamental issues like poverty, healthcare and access to clean water. A critique made in the global south is that the SDGs are tokenistic and superficial (Amorós Molina et al., 2023). As a result, SDG integration may be less emphasised or seen as less urgent in these contexts, despite the global importance of the goals. In the global north ‘minority’ world too, there is resistance to uptake of the UN SDGs. The DESNZ Public Attitudes Tracker in the Spring of 2024 revealed that 18% of people in the UK reported being either not very or not at all concerned about climate change (UK Government, 2024). These voices, although increasingly isolated, continue to challenge the relevance and necessity of the SDGs, dismissing them as ‘idealistic’ or irrelevant to certain academic disciplines.

This resistance is not just a challenge to educational reform; it is a direct confrontation with the urgent reality of global challenges like climate change, inequality and public health. SDG integration in education is not just about a curricular shift – it is far more fundamental, arguably to the continued survival of the planet. If SDGs are pushed to the margins or dismissed, we risk failing to equip future generations with the knowledge and skills needed to tackle the unprecedented challenges they will face.

Research shows, and our results indicate, that making educational change will meet challenges such as curriculum constraints, lack of faculty training, and the need for discipline‐specific content, resulting in a slow process toward reform (Fullan, 2007). Educational reforms, particularly those that require shifting perspectives and changing established teaching practices, typically face resistance, as sustainable reform is not just about introducing new content but also about altering the culture and mindset of the institution, including both faculty and students. In the case of SDG integration, this means providing not only resources and professional development but also creating a culture of collaboration where educators are encouraged to see SDGs as integral to their teaching rather than as an additional burden (Fullan, 2007).

In the face of resistance, we must recognise that integrating SDGs into the curriculum is not just about adding new content – it is about fostering a shift in how we educate. Overcoming opposition to SDG integration requires not just incremental changes in educational content, but a deep cultural transformation within institutions, some of which are rising to the challenge (Advance HE, 2025; Queen's University Belfast, 2024; The Physiological Society, 2023a, 2023b, 2024; United Nations, 2025).

### 4.6 Conclusion

This study underscores the critical importance of integrating sustainable development goals into Physiology education, highlighting that structured exposure to sustainability concepts significantly enhances student awareness and fosters a broader understanding of the intersection between human health, environmental sustainability, societal and global well‐being. Given the escalating global health and environmental challenges, embedding sustainable development within Physiology education has never been more essential. Higher education institutions must take a proactive role in preparing future professionals who can tackle complex, interdisciplinary challenges with informed, evidence‐based solutions.

Ultimately, SDG integration in education is not just about raising awareness – it is about transforming how students think, engage and apply knowledge to real‐world sustainability challenges. Overcoming barriers such as curriculum overload and lack of institutional support, providing tailored faculty development and overcoming opposing attitudes towards SDG integration will be crucial for sustained success.

Notwithstanding this, the rewards for such successful engagement are legion. Students educated in conjunction with the SDGs gain a sense of purpose and meaning, potentially addressing mental health challenges seen in many third level institutions. By preparing young adults to address the global challenges of the 21st century, higher education institutes have the potential to drive sustainable development by equipping future professionals with the critical thinking skills and knowledge required to build an equitable and sustainable future.

## AUTHOR CONTRIBUTIONS

*Conception and design of the work*: Mary McGahon, Clare Foy, Sean Roe. *Acquisition, analysis or interpretation of data for the work*: Mary McGahon, Sarah Geraghty. *Drafting the work or revising it critically for important intellectual content*: Mary McGahon, Sarah Geraghty, Clare Foy, Sean Roe. All authors approved the final version of the manuscript, agree to be accountable for all aspects of the work in ensuring that questions related to the accuracy or integrity of any part of the work are appropriately investigated and resolved and qualify for authorship. Only those authors who qualify for authorship are listed.

## CONFLICT OF INTEREST

The authors declare they have no conflicts of interest.

## FUNDING INFORMATION

No funding has been received for this work.

## Data Availability

The raw, anonymised data are available upon request to the corresponding author.

## APPENDIX 1

### 1.1

**TABLE A1:** Summary data assessing awareness and understanding pre‐ and post‐assignment.

Measure	Pre‐assignment awareness of SDGs	Post‐assignment awareness of SDGs	Pre‐assignment understanding of SDGS	Post‐assignment understanding of SDGS
Mean	7.02	8.33	5.78	7.87
SD	2.07	1.44	2.02	1.62
SEM	0.29	0.20	0.28	0.23
*n*	51	52	51	52
Lower 95% CI of mean	6.44	7.93	5.22	7.41
Upper 95% CI of mean	7.60	8.73	6.35	8.32

**TABLE A2:** Statistical analysis of awareness and understanding pre‐ and post‐assignment.

Dunn's multiple comparisons test	Mean rank 1	Mean rank 2	Mean rank diff.	*Z*	Adjusted *P‐*value
Pre‐awareness of SDGs versus post‐awareness of SDGs	94.93	136.50	−41.61	3.594	0.0013
Pre‐understanding of SDGS versus post‐understanding of SDGS	60.28	121.30	−60.97	5.267	<0.0001
Pre‐awareness of SDGs versus pre‐understanding of SDGS	94.93	60.28	34.65	2.979	0.0116
Post‐awareness of SDGs versus post‐understanding of SDGS	136.50	121.30	15.29	1.327	0.7378
